# Naturally occurring lysosomal storage disease consistent with neuronal ceroid lipofuscinosis in a group of 5 related captive rhesus macaques (*Macaca mulatta*)

**DOI:** 10.1177/03009858261445995

**Published:** 2026-05-12

**Authors:** Katherine Olstad, Gabrielle Pastenkos, Javier Barrera-Zarate, Sreetharan Kanthaswamy, Rebecca Sammak, Marie-Josee Lemoy

**Affiliations:** 1 University of California, Davis, Davis, CA

**Keywords:** autosomal recessive inheritance, lysosomal storage disease, neurodegeneration, neurologic, neuronal ceroid lipofuscinosis, nonhuman primate, rhesus macaque

## Abstract

Lysosomal storage diseases (LSDs) are a diverse group of inherited disorders characterized by the accumulation of undegraded macromolecules within lysosomes, leading to cellular dysfunction and progressive organ damage. Neuronal ceroid lipofuscinosis (NCL) is a subset of LSDs defined by the accumulation of autofluorescent lipopigments in neurons and, in some cases, peripheral tissues. While naturally occurring LSDs have been documented in domestic and laboratory species, reports in nonhuman primates (NHPs) remain rare. This study describes an LSD in 5 related rhesus macaques (*Macaca mulatta*) at the California National Primate Research Center, with clinical and histopathologic features supportive of NCL. Affected animals exhibited progressive cognitive and motor deficits, along with an atypical musculoskeletal phenotype including coarse facial features. Histologic examination revealed neuronal cytoplasmic vacuolation, primarily in the cerebellum and brainstem, with storage material exhibiting autofluorescence, positive staining with Sudan black, and luxol fast blue, and a positive periodic acid-Schiff reaction. Transmission electron microscopy identified structures resembling curvilinear inclusions and granular osmiophilic deposits, supporting a NCL diagnosis. This report expands the spectrum of NCL presentations in NHPs, reinforcing their potential as an alternative model for LSD research. The identification of this naturally occurring NCL model in NHPs may enhance our understanding of translational models for therapeutic strategies.

Lysosomal storage diseases (LSDs) represent a group of over 70 disorders characterized by the accumulation of nondegradable macromolecules within lysosomes.^5^ This accumulation arises from mutations in genes encoding lysosomal enzymes, transporters, or activator proteins, which lead to absent, reduced, or altered enzyme function. Consequently, substrates that would typically be degraded within lysosomes accumulate, disrupting cellular homeostasis and causing cellular dysfunction and death.^5,17^ Based on the type of stored material, LSDs are classified into 4 main categories: sphingolipidoses, mucopolysaccharidoses, glycogenoses, and glycoproteinoses.^17^

The clinical presentation of LSDs is highly heterogeneous, often involving multiple organ systems, but with 1 system typically predominating. Clinical manifestations and age of onset vary widely, from prenatal to late adulthood, depending on the specific enzyme affected, the accumulated substrate, and the tissues involved. Neurological impairment is a common feature in approximately two-thirds of all human LSDs,^16^ with symptoms including cognitive impairment, motor dysfunction, seizures, and neurodegeneration.^17^ In human adult-onset cases, neurological symptoms may be more subtle, such as depression, dementia, or psychosis.^16^

Most LSDs follow an autosomal recessive inheritance pattern, with a few exceptions, such as Fabry disease and Hunter syndrome (MPS II), which are X-linked.^5^ Although the overall prevalence of LSDs in humans is estimated at approximately 1 in 5000, individual disorders are much rarer, occurring in 0.2 to 2.5 per 100 000 individuals.^17^

Naturally occurring LSDs have been documented in several domestic and laboratory species, including dogs, cats, horses, and mice.^9^ Mouse models have been instrumental in understanding the pathogenesis of LSDs and evaluating treatment modalities.^16,17^ However, these models, predominantly inbred knockout mice, may oversimplify the complexity of LSDs, and findings from treatment studies often fail to translate effectively to clinical trials.^8^ The genetic heterogeneity of nonhuman primates (NHPs) compared with inbred mice and their neuroanatomical similarities to humans offer a promising alternative model, though reports of LSDs in these species also remain rare. Notable examples include spontaneous *CLN7* and *CNL2* mutations linked to neuronal ceroidlipofuscinosis (NCL) in a group of Japanese macaques (*Macaca fuscata*) at the Oregon National Primate Research Center (ONPRC) and cynomolgus macaques (*Macaca fascicularis*) at the Tsukuba Primate Research Center (TPRC), respectively, as well as a globoid cell leukodystrophy (Krabbe disease) model in rhesus macaques (*Macaca mulatta*) at the Tulane Primate Center.^3,11,13,21^

This report builds on the limited documentation of naturally occurring LSDs in NHPs by describing the clinical and histological findings of LSD in 5 related rhesus macaques at the California National Primate Research Center (CNPRC). These cases share similarities with the NCL documented in Japanese macaques at the ONPRC and cynomolgus macaques at TPRC, underscoring the importance of investigating such spontaneous models for diagnostic and research purposes. By detailing these findings, this case series further bridges the gap between clinical observations and the exploration of NHPs as viable alternatives to traditional models for LSD research.

## Materials and Methods

### Case Selection

All animals in this study were housed at the CNPRC and were cared for in compliance with the Animal Welfare Act and the *Guide for the Care and Use of Laboratory Animals*.^14^ Cases were retrospectively identified from the CNPRC database between 1991 and 2011. The selection criteria were based on clinical signs observed in a key affected animal (case 1), first identified in 2011. This animal exhibited clinical features, including cognitive deficits and motor dysfunction, serving as the initial case inclusion benchmark.

Case 1 was used to construct a detailed pedigree spanning 20 years and 4 generations. The clinical histories of all animals in the pedigree were reviewed for similar clinical signs and phenotypes. Animals meeting the basic inclusion criteria underwent further retrospective histologic evaluation of available tissues, focusing on central nervous system tissues, such as the brain and spinal cord, to confirm if any pathological changes consistent with the observed clinical signs were present.

### Gross and Histologic Evaluation

Necropsies were performed on all animals at the CNPRC by pathologists who specialize in NHPs. All available tissue sections were processed routinely, sectioned at 5 µm, and stained with hematoxylin and eosin. Formalin-fixed, paraffin-embedded tissues were additionally processed for histochemistry on select brain, spinal cord, heart, retina, and gastrointestinal sections following standard laboratory procedures for luxol fast blue (myelin), Sudan black (neutral fats and phospholipids), and periodic acid-Schiff (PAS) with and without diastase (to differentiate glycogen from other PAS-positive elements). In addition, a fluorescence microscope was used to assess autofluorescence of storage material on hematoxylin and eosin-stained sections.

### Transmission Electron Microscopic Evaluation

Brain and spinal cord tissue samples that had been fixed in 10% neutral-buffered formalin were trimmed into 2 mm pieces and postfixed in 1% osmium tetroxide in 0.1 M sodium cacodylate buffer for 2 hours. The tissues were then serially dehydrated in ethanol, infiltrated with propylene oxide and embedded in resin in plastic molded embedding capsules. Sections were obtained using an ultramicrotome. Semithin (1 μm) sections were stained with 1% toluidine blue and examined to identify areas of interest. Ultrathin (90 nm) sections were cut from those areas. The sections were mounted onto 300-mesh copper grids, stained with uranyl acetate and lead citrate, and observed under a transmission electron microscope.

## Results

### Signalment

**Figure 1.**
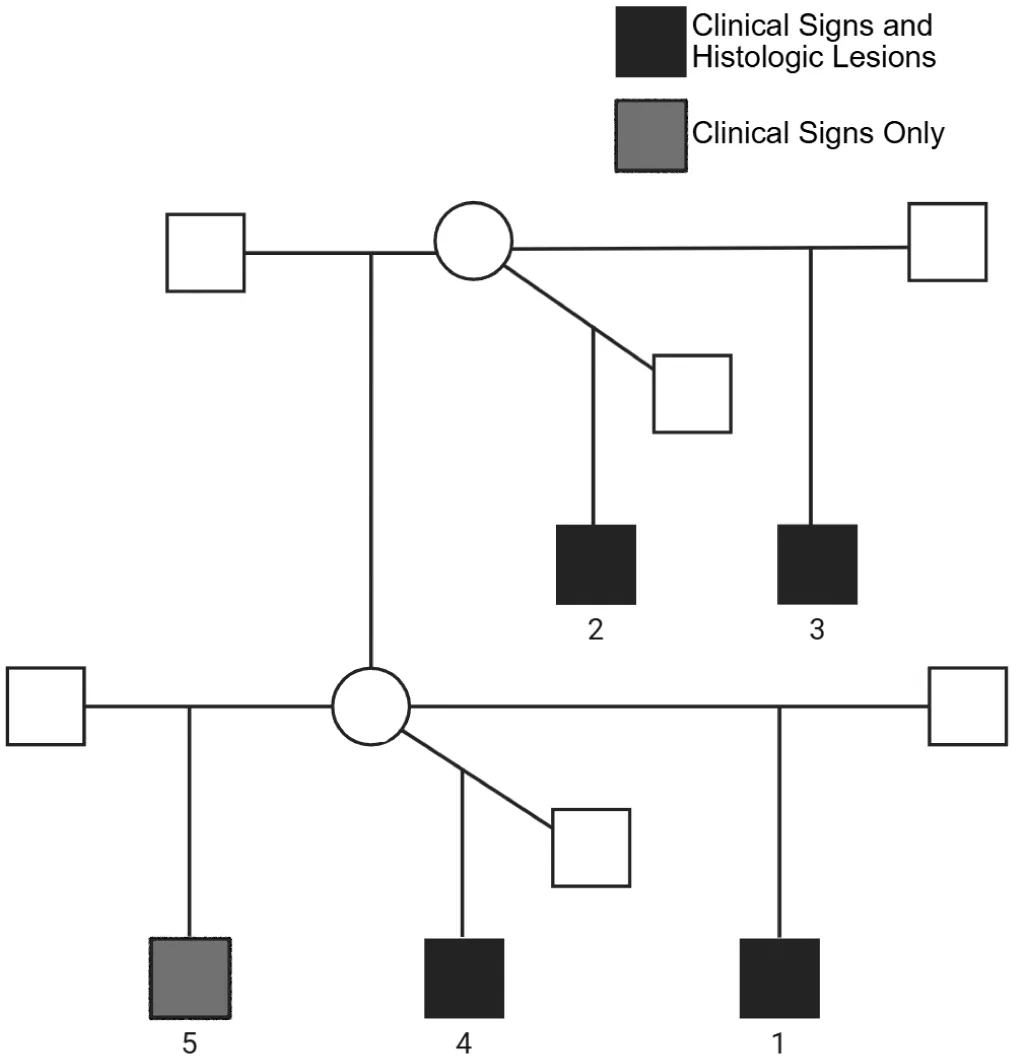
Pedigree of 5 related male rhesus macaques (cases 1-5) exhibiting similar neurologic clinical signs. Circles represent females and squares represent males. Open circles and squares indicate unaffected animals. Solid black squares represent animals with both clinical signs and histologic lesions supportive of a lysosomal storage disorder. Gray squares indicate an animal presenting with neurologic clinical signs, but tissues from the central nervous system were unavailable for histologic examination.

Five related male rhesus macaques were identified based on the established case criteria and pedigree analysis (Fig. 1). The inbreeding coefficient was not significant as 2 of the sires (sires of animals 2 and 3) within the pedigree were unknown, but the kinship coefficient of affected animals was either 0.625 or 0.125. All animals were considered clinically and phenotypically normal at birth. The age of onset for clinical signs ranged between 1 and 3 years (average, 1.6 years) (Table 1).

**Table 1.** Clinical characterization of phenotype.

Case	Age at clinical observation	Clinical phenotype observed
1	3-5 years	Abnormal gait, prominent brow, broad nose, short neck, thick limbs and digits, immature dentition, macroglossia, kyphosis
2	1-4 years	Bilateral tibial valgus, kyphosis, disuse of hands, macroglossia
3	1-4 years	Abnormal gait, intermittent lameness, short neck, short limb, joint laxity, large head, bilateral patellar luxation
4	1 year	Hunched posture, shortened long bones, brachycephaly, hindlimb weakness with poor range of motion
5	2-5 years	Abnormal gait, abnormal facial structure, immature dentition, gingival hyperplasia, hepatosplenomegaly

### Clinical Presentation

**Figure 2.**
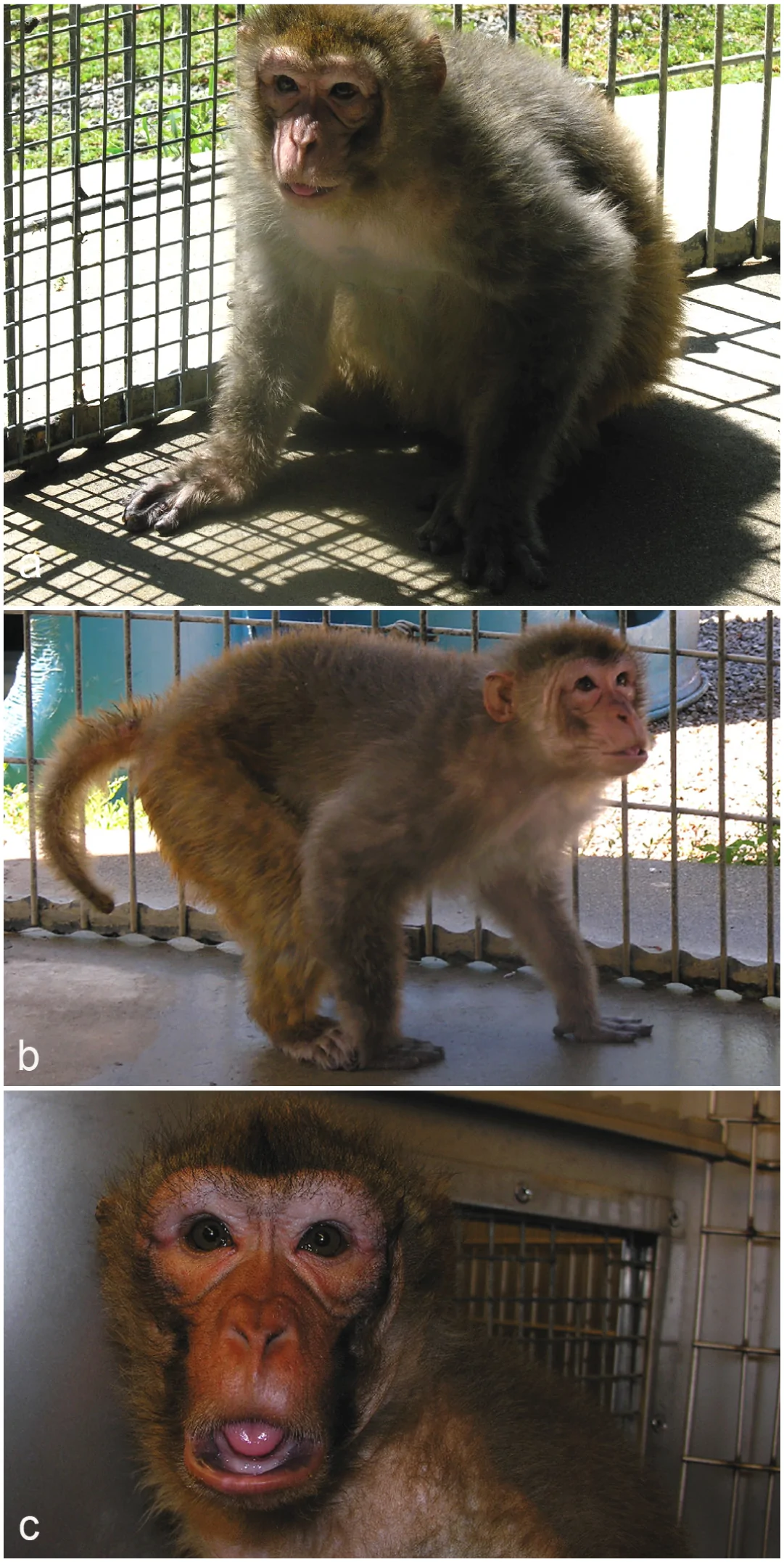
Case 1 with the most prominent clinical phenotype of the group exhibiting abnormal facial and musculoskeletal features. (a) Short neck and thick limbs and digits. (b) Abnormal hunched walking posture. (c) Prominent brow ridge, broad nose, immature dentition, and macroglossia.

Clinical signs included varying severity of progressive cognitive deficits such as difficulty learning new tasks and an inability to groom properly. All animals exhibited some degree of an abnormal musculoskeletal phenotype including abnormal gait, and coarse facial features such as a broad nose, thick skull, prominent brow, macroglossia, short neck, and thick limbs (Fig. 2, Table 1).

As this is a retrospective study, each animal was clinically observed at different time points between 1991 and 2011. Consequently, ancillary testing varied between individuals based on their clinical presentation rather than being part of a standardized routine testing protocol. Case 1 received a gated computed tomography scan of the extremities directly prior to the necropsy, which revealed no major bone anomalies. Case 2 underwent electromyography testing, which revealed mild widespread abnormalities particularly affecting the shoulder region and biceps brachii, but nerve conduction was within normal limits. Enzymatic testing was not performed on any of the animals as a storage disorder was not considered at the time of the work-up. Euthanasia was elected in all cases due to the progressive worsening of locomotor symptoms.

### Gross and Histologic Findings

**Figure 3.**
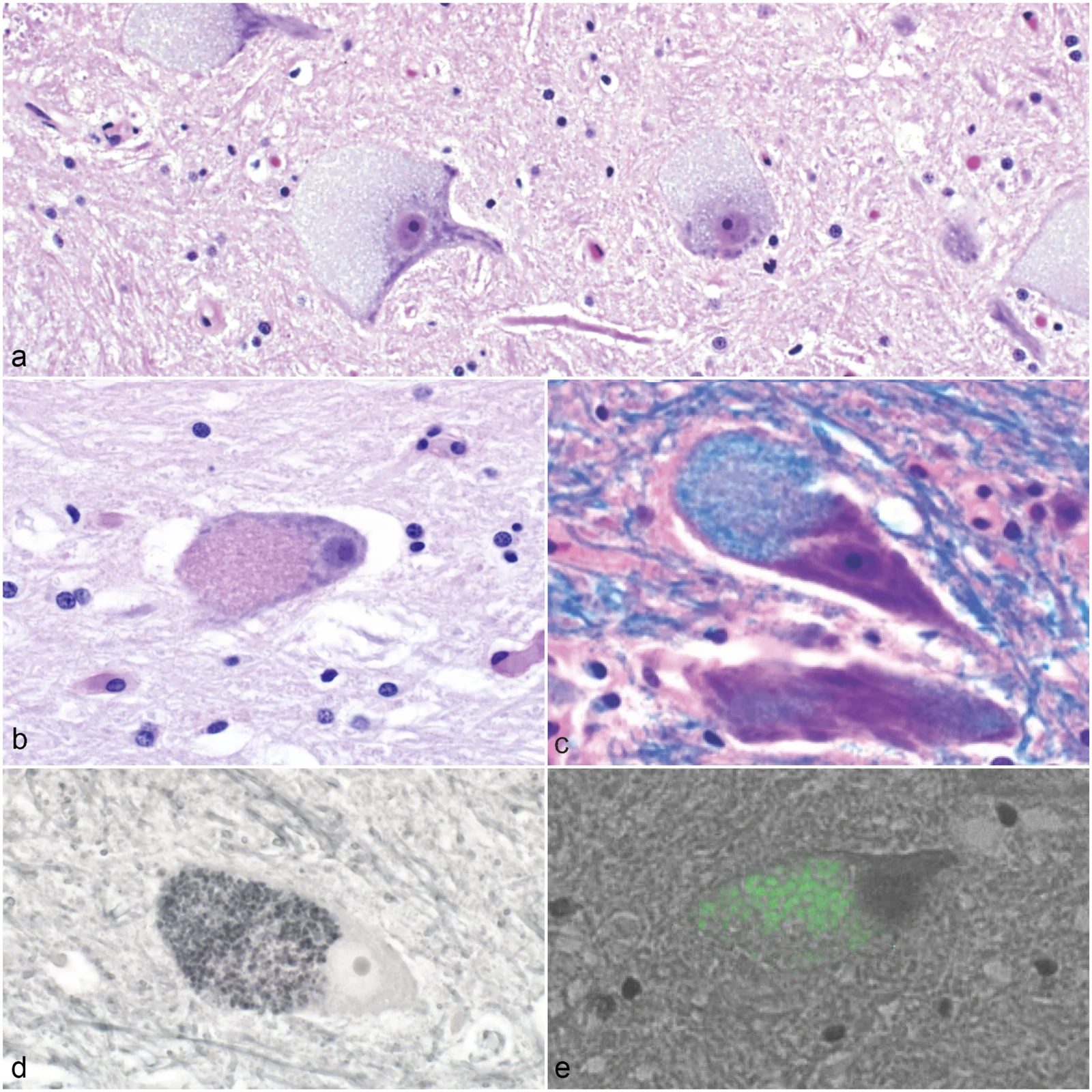
Microscopic features of the neuronal storage material in the cerebrum of case 1. (a) Multiple neurons are swollen 2 to 3 times normal size. The nuclei are displaced peripherally, and the cytoplasm is expanded by numerous 2 µm diameter, clear to slightly eosinophilic vacuoles displacing Nissl substance. Hematoxylin and eosin. (b-d) Neuronal vacuoles express a positive reaction with (b) periodic acid-Schiff with and without diastase (the latter not shown here), (c) luxol fast blue, and (d) Sudan Black. (e) The vacuoles are autofluorescent in ultraviolet light.

**Figure 4.**
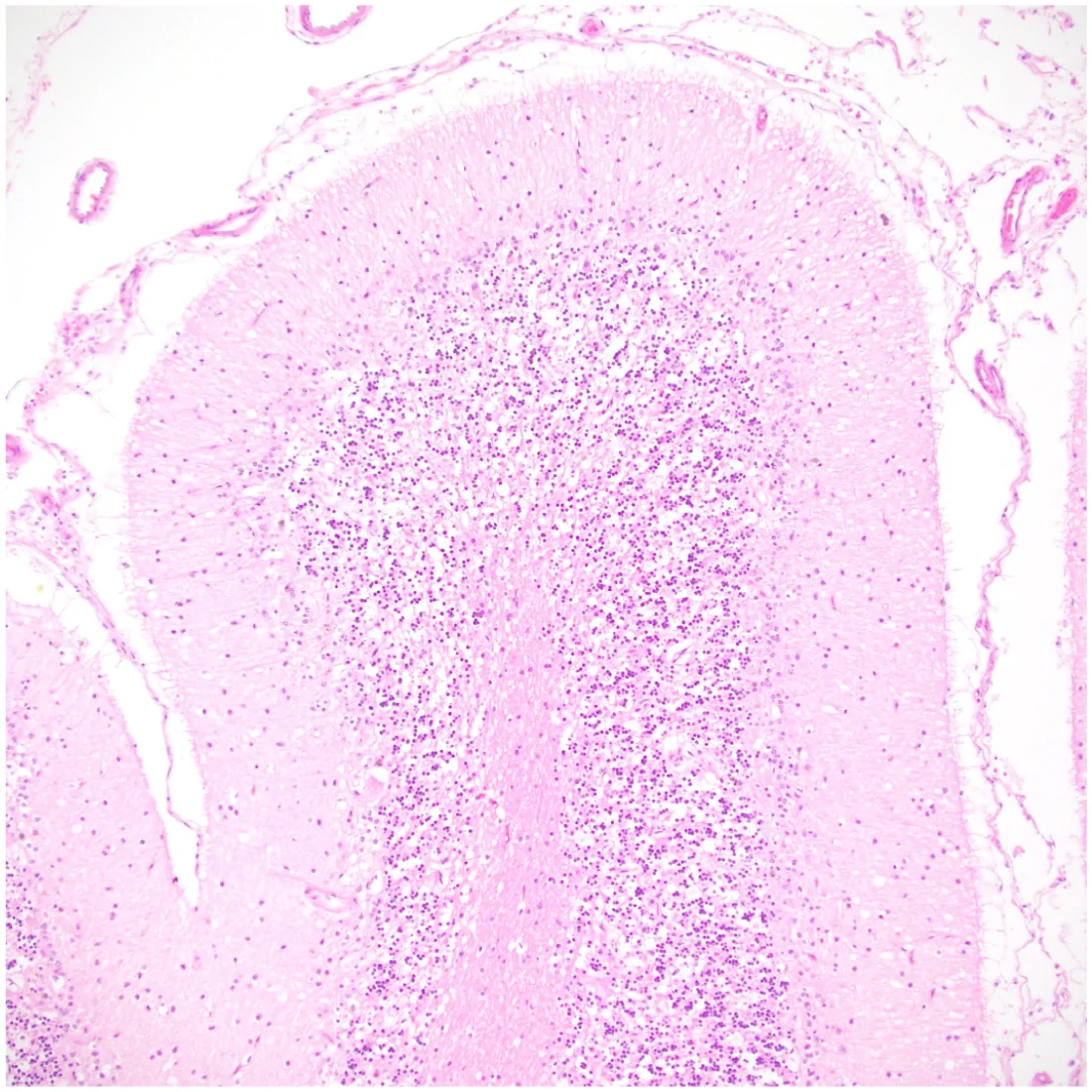
Decreased cellularity and blurring of the molecular and granular layers of the cerebellum. Hematoxylin and eosin.

All animals exhibited mild-to-moderate musculoskeletal abnormalities consistent with the previously described clinical phenotype (Table 1). Archival tissues were available for histologic examination in 5 of 5 animals, and included brain (4/5), spinal cord (1/5), heart (3/5), eye (3/5), bone (1/5), and gastrointestinal tract (3/5). The primary histologic lesion in all cases with available brain and spinal cord was neuronal cytoplasmic swelling, accompanied by variable neuronal loss and gliosis in the central nervous system. Affected neurons were up to 3 times their normal size due to cytoplasmic accumulation of numerous, 2 µm diameter, clear to weakly eosinophilic vacuoles with peripheral displacement of Nissl substance and of the nuclei (Fig. 3a). The cytoplasmic vacuoles were variably positive by PAS reaction (Fig. 3b), resistant to diastase, and strongly positive when stained with luxol fast blue (Fig. 3c) and Sudan black (Fig. 3d). Storage material was autofluorescent when exposed to ultraviolet light (Fig. 3e). In the most severe case (case 1), affected neurons were found in the following locations: cerebral subcortical nuclei (thalamus, hypothalamus, basal ganglia), cornu ammonis regions of the hippocampus (specifically the pyramidal neurons), the cerebellar cortex (Purkinje cell layer), deep cerebellar nuclei, brainstem nuclei, and spinal cord gray matter (decreasing prevelence from ventral horns > intermediate zone > dorsal horns). Additional lesions consisted of thinning of the cerebellar molecular layer, Purkinje cell loss, reduced cellularity of the granular layer, and patchy loss of distinction between molecular and granular layers (Fig. 4). The last 2 changes appeared more pronounced medially/centrally (toward the vermis), but this finding is interpreted with caution due to limited tissue availability. Findings were similar but less severe in cases 2 to 4, with affected neurons most prominent in the cerebellar cortex and brainstem. Minimal, sporadic, perineuronal glial satellitosis (cases 1, 2, and 4) and an isolated microglial nodule (case 2) were observed in the medulla oblongata, but gliosis was not a prominent feature. Neurons with cytoplasmic accumulation of vacuolated material identical to those described in the central nervous system were identified in submucosal and/or myenteric ganglia in 3/3 animals for which gastrointestinal tissue was available. In some ganglia, all neurons visible in the section were affected; in other ganglia, affected neurons were mixed with variable numbers of histologically normal neurons. The brain, spinal cord, and gastrointestinal tract were not available for evaluation from case 5. Lesions unrelated to storage disease included chronic arthritis (3/5), cruciate ligament rupture (1/5), gingival hyperplasia (3/5), heart lesions (4/5), splenomegaly and hepatomegaly (2/5), and colitis (1/5) (Table 2).

**Table 2.** Significant gross and histologic lesions.

Case	Tissues with vacuolar change consistent with storage material	Additional necropsy findings
1	Brain, spinal cord, myenteric ganglia (stomach, colon, rectum), submucosal ganglia (colon, rectum)	Left atrioventricular endocardiosis, semilunar valvular thickening, small cervical spinal cord, aspiration pneumonia
2	Brain (spinal cord not available), myenteric ganglia (stomach, duodenum, colon), submucosal ganglia (colon)	Myocardial fibrosis, colitis, arthritis, splenomegaly and hepatomegaly (amyloid), gingival hyperplasia
3	Brain (spinal cord not available), myenteric and submucosal ganglia (stomach, cecum, colon)	Myocardial hypertrophy and fibrosis, arthritis, gingival hyperplasia
4	Brain (spinal cord not available)	Meningeal pigment (melanin) and edema (only brain available)
5	(Brain and spinal cord not available)	Left atrioventricular endocardiosis, choledochitis, right cruciate ligament rupture, hepatosplenomegaly of unknown cause, gingival hyperplasia

### Transmission Electron Microscopy Findings

**Figure 5.**
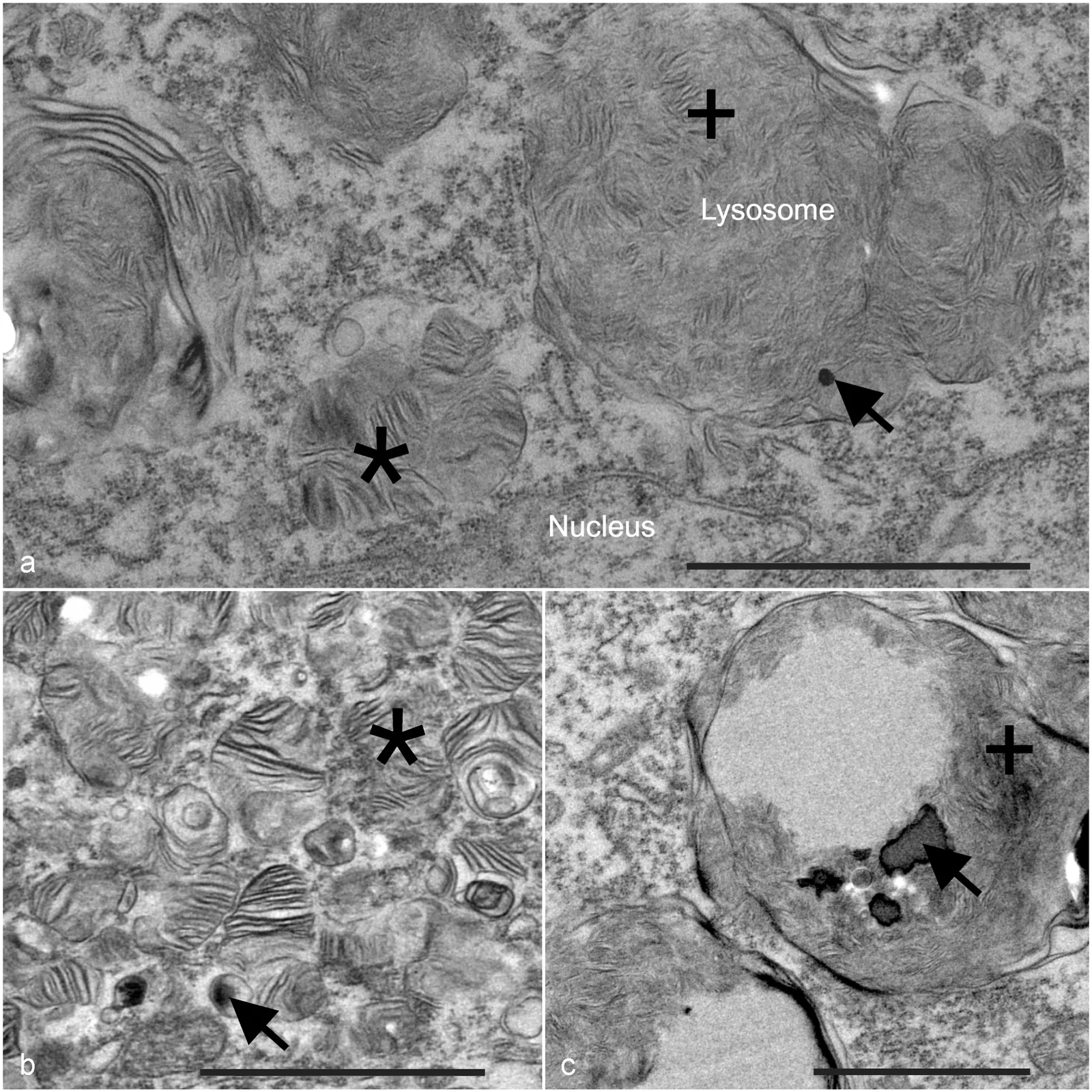
Ultrastructural features of the lysosomal storage material within the brainstem of case 1. (a) Neuron with large numbers of membrane bound, tightly packed, concentric or lamellated fingerprint-like structures (asterisk) or electron-dense stacks of alternating short, curved membrane bound curvilinear-like structures (plus sign) within lysosomes, which expand most of the cytoplasm. The engorged lysosomes abut and slightly displace the nucleus. There is a single ovoid electron-dense structure consistent with a granular osmiophilic deposit (GROD) (arrow). Scale bar, 2 µm. (b) In some regions, the lysosomal storage material is predominantly fingerprint-like structures (asterisk) with fewer variably sized accumulations of GRODs (arrow) within lysosomes. Scale bar, 2 µm. (c) A larger GROD structure (arrow) adjacent to curvilinear-like structures (plus sign) within a lysosome. Scale bar, 2 µm.

Ultrastructural examination of the neurons within the cerebrum and spinal cord revealed that the intracytoplasmic storage bodies observed histologically were membrane-enclosed structures within lysosomes containing a variable and mixed accumulation of materials. These bodies were composed of one or more of the following components: (1) structures resembling granular osmiophilic deposits (GRODs), which varied in size and were oval to round and highly electron-dense (Fig. 5a–c); (2) Curvilinear profiles, consisting of short, thin, curved lamellar stacks that alternated between low and high electron densities (Fig. 5a, c); (3) Fingerprint profiles, made up of tightly packed concentric membranous layers (Fig. 5a–c).

## Discussion

Diagnosing LSD’s in NHPs is particularly challenging, as an accurate antemortem diagnosis in human medicine relies on specialized biochemical and molecular genetic tests, which are both sophisticated and highly specific. Commonly used screening tests are designed for human newborns and can be influenced by factors such as medications, physiological status, and age.^2^ In our case series, all subjects were considered clinically normal at birth, and due to the low prevalence of LSDs in NHPs, these disorders are not included in CNPRC’s routine newborn screening procedures. The retrospective nature of this study further complicated the diagnosis, as LSDs were not initially considered in the clinical differential diagnosis for each individual presentation, which spanned decades. The progressive neurodegeneration and phenotype of the most severely affected animal (case 1) ultimately prompted further investigation, leading to a retrospective review of the animal’s pedigree and additional evaluation of brain sections. This analysis was not historically part of the standard pathology work-up. It was only at this stage that a familial storage disorder was first considered, leaving limited postmortem resources available for definitive confirmation.

Based on pedigree analysis and the clinical and pathological presentation of the 4 related male rhesus macaques (cases 1-4), including transmission electron microscopy, a primary inherited storage disorder supportive of neuronal ceroid lipofuscinosis was diagnosed. Given the shared phenotype and familial relationship within the CNPRC cohort, the same diagnosis is presumed for case 5 despite the lack of histological confirmation. Similar clinical cases in NHPs have been reported, including 6 Japanese macaques at the ONPRC and 3 cynomolgus macaques at the TPRC.

Neuronal ceroid lipofuscinosis are a group of recessively inherited, progressive neurodegenerative diseases collectively referred to as Batten diseases, characterized by the accumulation of autofluorescent lipopigment within the central nervous system and, in some cases, peripheral tissues such as the heart and retina.^1,11^ In humans, these disorders primarily manifest in childhood and are caused by mutations in at least 13 different *CLN* genes.^1,18^ The *CLN* gene family is functionally diverse, and while the precise molecular roles of many individual CLN proteins remain incompletely defined, they are generally implicated in lysosomal integrity, protein and lipid degradation, autophagy, endomembrane trafficking, and cellular homeostasis.^10^ Despite genetic heterogeneity, all forms of NCL share a defining pathological feature of intracytoplasmic accumulation of autofluorescent lipopigment consistent with the findings in this study. To complement the presence of autofluorescence, transmission electron microscopy can serve as a valuable diagnostic tool, particularly in less-than-ideal circumstances when enzymatic testing is unavailable, as in this case. However, storage disorders can be complex, leading to diverse storage accumulations depending on the specific disease. In NCL, lipopigment deposits identified by electron microscopy are classically categorized as either GRODs seen commonly with *CLN1* and *CLN10* mutations or non-GROD patterns seen commonly in *CLN2* mutations. A mixed pattern can also be seen in the remaining CLN forms.^1^ Non-GROD structures include curvilinear and lamellar profiles, all of which were observed in this study in addition to GRODs, further supporting the diagnosis of NCL.^18^

A subclassification of NCL has been established in humans based on the age of onset and associated gene mutation: congenital (mutations in *CLN10)*; infantile, between 6 and 18 months of age (mutations in *CLN1, CLN10*, and *CLN14)*; late-infantile, between 2 and 5 years of age (mutations in *CLN1, CLN2, CLN5, CLN6, CLN7*, and *CLN8)*; classic juvenile, between 3 and 7 years (mutations in *CLN1, CLN3*, and *CLN5)*; late form of juvenile, between 8 and 18 years (mutations in *CLN6, CLN10*, and *CLN12);* and adult (mutations in *CLN4*, *CLN11, CLN12*, and *CLSN 13)*. Most childhood-onset forms are characterized by progressive cognitive and motor decline, vision loss, seizures, and premature death, whereas the rarer adult-onset forms primarily manifest as dementia.^3,10,15^

Applying the human subclassification of NCL to NHPs requires consideration of species-specific differences in aging. Macaque species age approximately 3 times faster than humans, with full weaning occurring around 1 year of age and puberty between 2.5 and 4.5 years.^20^ Accordingly, macaque age classification includes infants (<1 year), juveniles (1-4 years), and adults (>5 years).^2,6^ In this study, the average age at onset of noticeable clinical signs was 1.6 years ranging from 1 to 3 years. This pattern aligns with the classical juvenile-onset classification of NCL described in humans.^15^ Similarly, the 3 animals identified at TPRC had an average onset of 2.2 years with a range of 1.9 to 2.5 years, which also falls within the classical juvenile form.^13^ In contrast, animals at ONPRC with clinical signs exhibited a slightly older onset, averaging 4.4 years with a range of 4.5 to 5.5 years of age (late juvenile subclassification), with one animal presenting at 1.5 years (classic juvenile subclassification).^11^ The younger onset observed at the CNPRC and TPRC compared with the majority at the ONPRC may be attributable to differences in observational methods, such as indoor housing versus initial assessments conducted from a distance in a 1-acre field cage. Despite the age of onset, all animals at all locations reached humane endpoints based on clinical welfare concerns due to the progressive nature of the disease, which, on average, is around 3 to 6 years of age. Interestingly, the ONPRC study, which screened their colony, did not find any homozygous animals over the age of 6 suggesting their form of NCL is highly fatal at a young age.^11^

All known NCL-associated genes are located on autosomes and are typically inherited in an autosomal recessive pattern.^12^ *CLN7* and *CLN2* gene mutations at the ONPRC and TPRC, respectively, were identified and consistent with this inheritance pattern.^11,13^ The role of CLN7 is not entirely understood but is implicated in lysosomal transport pathways while CLN2 plays a role in protein degradation pathways.^11,13^ Interestingly, both genes are associated with the late-infantile subclassification of NCL in humans, which typically manifests between 2 and 5 years of age; equivalent to approximately 6 weeks to 1 year in macaques.^12^ This discrepancy between gene identification and age of onset suggests that human subclassifications may not directly align with those in NHPs. Alternatively, early cognitive and behavioral changes in younger macaques may have gone undetected, as these signs can be more subtle in NHPs. It’s also important to point out that age extrapolations are estimates and may not be entirely accurate for fitting NHPs into these general categories. Given that all affected animals in this current study were male, an X-linked inheritance pattern was initially considered; however, male predominance is more likely a consequence of the small sample size, similar to what was observed in the TPRC cohort, who also had a male predominance. Due to the retrospective nature of this study, additional genetic testing to determine the precise mutation was not performed.

Imaging findings in the ONPRC and TPRC cohorts revealed pronounced cerebellar depletion, with a particular loss in the granular and Purkinje cell layers.^11,13^ This pattern closely aligns with the histopathological findings in this study, where Purkinje cells were more severely affected than cerebral neurons. Unfortunately, no advanced antemortem imaging of the brain was performed, thus additional and more subtle comparisons observed on brain scans between the cohorts is not possible.

Although accumulation of storage material in extra-cerebral organs is rare in NCLs, lesions are reported in the gastrointestinal ganglia, retina, and cardiac conduction system, with involvement of the latter potentially leading to fatal arrhythmias.^1^ Enteric neurons in cases 1, 2, and 3 contained storage material with a similar phenotype as described in the central nervous system, including autofluorescence. Blindness was not documented as a clinical sign in any of the 5 animals. In addition, in the 2 cases where the retina was available for examination (cases 1 and 2), all neuronal layers were preserved and retinal ganglion cells lacked PAS-positive storage accumulations as well as autofluorescence. This suggests that ocular tissues are unaffected in this disorder. In this study, clinical signs of cardiac disease were not detected, but cardiac abnormalities were noted postmortem in 4 animals (cases 1, 2, 3, and 5) which included valvular lesions (cases 1 and 5) and myocardial fibrosis (cases 1, 2, 3, and 5). As with central nervous system lesions, case 1 exhibited the most severe changes consisting of severe thickening of the left-sided atrioventricular and aortic semilunar valve leaflets, characterized by disorganization and accumulation of round cells with abundant foamy cytoplasm. Cytoplasmic material was PAS-negative and lacked intrinsic autofluorescence. Valvular lesions in case 5 were similar although less severe, with accumulation of foamy round cells limited to the left atrioventricular valvular leaflets. Myocardial fibrosis was most severe at the insertion site of the valvular leaflets but could also be seen multifocally dissecting throughout the myocardium. As these animals were decades younger than the age at which these changes typically manifest, the possibility of localized storage product accumulation was considered. The foamy cells, however, were PAS-negative, lacked intrinsic autofluorescence, and were not distributed within the cardiac conduction system, as is reported in human NCL cases.

Although the histologic, histochemical, and transmission electron microscopic features described here are considered classic for NCL, particularly the autofluorescence of the storage material within neurons and presence of GRODs and curvilinear bodies on transmission electron microscopy, the musculoskeletal phenotype observed in this cohort is not characteristic of the disease and was not reported in either the ONPRC or TPRC studies. In this case series, all animals exhibited varying degrees of distinct coarse facial features, including prominent brows; a wide, broad nose with flaring nostrils; thickened lips; macroglossia; and musculoskeletal abnormalities such as kyphosis, short neck, and stocky stature. While motor deficits are common, the skeletal phenotype has not been reported in NCL and is more commonly associated with other LSDs, such as mucopolysaccharidosis or glycoproteinoses (including alpha-mannosidosis, fucosidosis, and galactosidosis), all of which exhibit a distinct histochemical profile from what is observed here.^4,7,19^ This striking discrepancy between the histopathologic findings and the observed musculoskeletal phenotype underscores the need for further genetic analysis in NHPs with suspected LSDs to define their disease spectrum and classification better. This cohort may represent a novel manifestation not yet described as NCL, which is a heterogenous disease with multiple genetic mutations leading to variable clinical presentations. However, without genetic testing, the possibility of an entirely different LSDs cannot be definitively excluded.

Overall, the findings in these rhesus macaques provide additional insights into the spectrum of NCL presentations in NHPs and highlight potential species-specific differences in clinical progression, phenotype, and organ involvement. It is recommended that brain tissue be included in routine workups, particularly in cases with musculoskeletal defects similar to those described here or long-term cognitive decline. Further recommendations for identifying naturally occurring storage diseases include collecting and preserving urine, either ante- or postmortem, for potential enzymatic testing and routinely freezing fresh liver for genetic analysis. Unfortunately, the CNPRC started routinely banking fresh-frozen liver in 2014, 3 years after case 1 was identified. Given that this is the third reported group of NHPs with the disorder, and that it manifests at a relatively young age, these cases reinforce the potential of NHPs as a valuable model for NCL. Their genetic diversity and neuroanatomical similarities to humans may help overcome some of the limitations seen in more traditional models.
